# Design and Experiment of Anti-Particle-Contamination MEMS Thermal Flow Sensor by Channel Geometry Optimization and Voltage Regulation

**DOI:** 10.3390/mi17101211

**Published:** 2026-10-21

**Authors:** Qilin Jiang, Jianfeng Wu, Junming Hou, Zhenxiang Yi

**Affiliations:** 1 School of Mechanical Engineering, Nanjing Institute of Technology, Nanjing 211167, China; jiangqilin@njit.edu.cn (Q.J.); hjunming@vip.163.com (J.H.); 2 Key Laboratory of MEMS of the Ministry of Education, Southeast University, Nanjing 210096, China; 220236252@seu.edu.cn

**Keywords:** MEMS thermal flow sensor, particle contamination, curved bypass channel, particle transport, electric-field regulation

## Abstract

Micro-electro-mechanical system (MEMS) thermal flow sensors are attractive for compact gas-flow measurement because of their small size, rapid response and low power consumption. However, under non-clean operating conditions, particles carried by the gas can enter the sensing region and progressively deposit on the chip, altering the local flow field and heat-transfer boundary conditions and thereby causing output drift and cumulative measurement error. To improve long-term stability in particle-laden environments, a MEMS thermal flow sensor was designed and implemented, and contamination was controlled along the particle pathway from transport toward the sensing region to deposition on the chip surface. The finite element method (FEM) simulation showed effective thermal isolation between the heating and temperature-sensing regions at ΔT = 20 °C, while the heating power increased monotonically with flow velocity in agreement with heat-loss theory. Particle-laden experiments indicated that the average measurement error was reduced by approximately 22.5% using the curved bypass channel for the flow speed up to 70 SLPM, with a more pronounced improvement at medium and high flow rates. For particles that still reached the vicinity of the chip, the surface electric field was further adjusted through the operating voltage. The sensor was tested under particle-filled flow for 2 h, and the results demonstrate that the contaminated regions were 14.34%, 10.40%, and 3.84% at supply voltages of 7, 8, and 9 V, respectively, indicating that lower operating voltage mitigated particle deposition.

## 1. Introduction

MEMS thermal flow sensors measure flow speed through convective heat transfer between microscale heating elements and the surrounding fluid. Their small size, fast response, ease of integration, and relatively low power consumption have enabled applications in industrial process control, environmental monitoring, medical-gas measurement, and microfluidic systems. Depending on the thermal excitation and readout mechanism, thermal flow sensors can operate in heat-loss, calorimetric, or thermal time-of-flight modes [1]. In recent years, improvements in chip-level thermal isolation, wide-range structures, and multimode readout have continued to enhance sensor performance [2,3]. Wide-range characterization and numerical optimization have further refined design methodologies [4,5], while intelligent estimation algorithms and novel sensing materials have been introduced to improve stability and sensitivity [6,7]. Thermal flow detection for harsh conditions, including high-temperature and high-speed environments, has also been advanced through sensitivity analysis and CFD-based parameter design [8,9], with increasing integration of intelligent and multi-parameter readout strategies [10].

Despite these advances, solid-particle contamination remains an important source of instability when thermal flow sensors operate for long periods in dusty gases or other non-clean environments. Particles entering the sensing region can collide with and adhere to the heater, temperature-sensing resistors, and adjacent surfaces. Progressive deposition changes the local velocity distribution and heat-transfer boundary conditions, causing the original flow–output calibration relationship to drift. Previous studies on calorimetric flow sensors have shown that both particulate contamination and oil-film deposition can produce appreciable measurement error and output drift [11,12]. Therefore, improving particle tolerance through modifications that do not substantially increase device volume or additional flow resistance is important for the engineering reliability of MEMS thermal flow sensors.

Consequently, in this study, a MEMS thermal flow sensor with a stable constant-temperature-difference (CTD) control mode was established so that contamination-induced deviations could be evaluated against a consistent clean-gas calibration. A curved bypass channel was then introduced to shift particles laterally before contact with the chip, thereby reducing the particle load near the sensing region. On this basis, for particles that still entered the chip vicinity, the operating voltage was reduced to weaken the local electric field and the electrostatic driving force. Thus, a voltage-adjustable operating method was further implemented to balance contamination mitigation and normal flow measurement. The improvements above are embedded directly in the original flow path and control electronics, which are evaluated in terms of flow-measurement error under particle-laden operation and the post-test surface-deposition state.

## 2. Principle and Method

### 2.1. Sensor Design and Implementation

**Figure 1:**
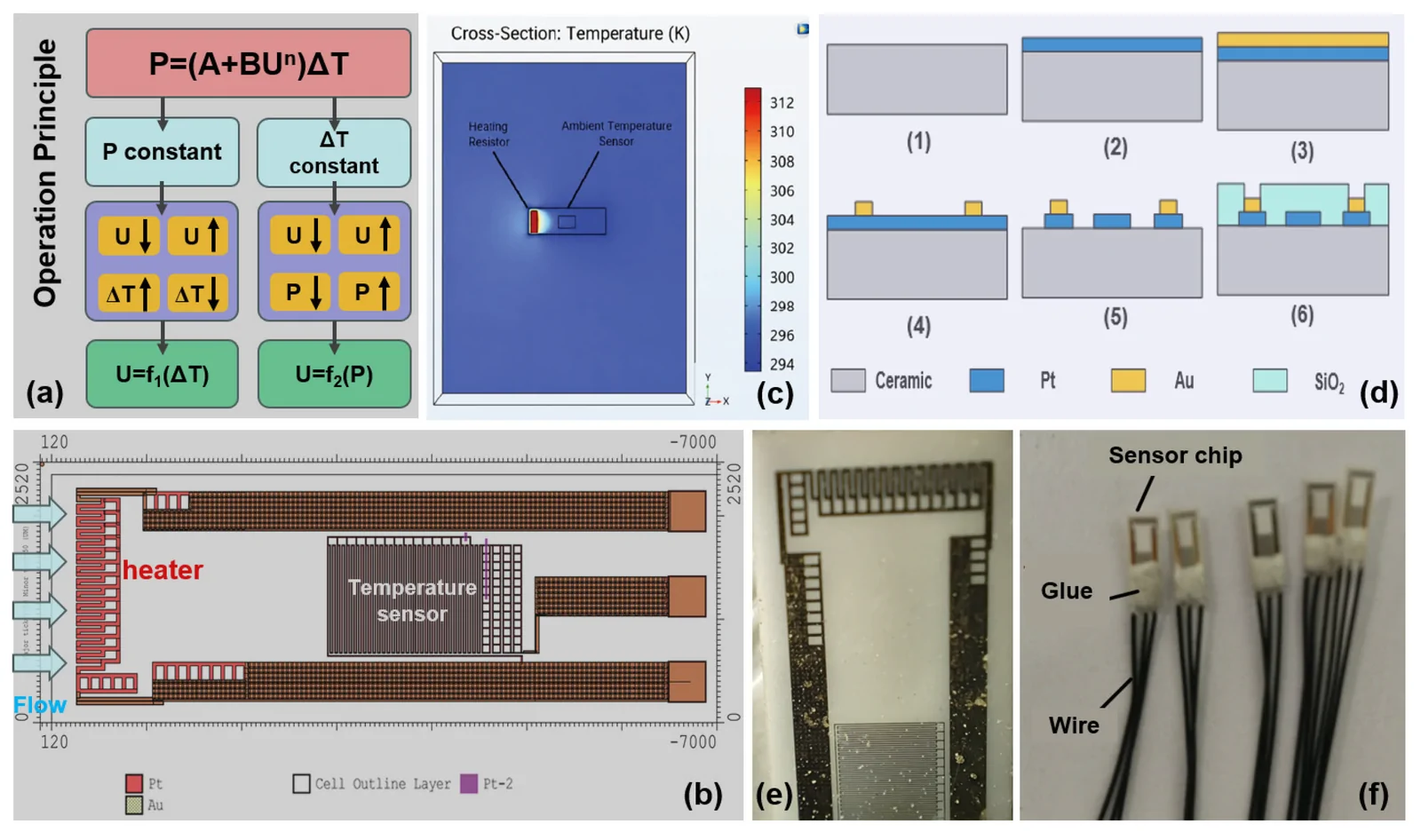
Design and implementation of the MEMS thermal flow sensor. (**a**) Principle of CTD operation, (**b**) FEM simulation, (**c**) layout of the sensor, (**d**) fabrication process, (**e**) photo of the fabricated sensor and (**f**) device after package.

A heat-loss principle was adopted for MEMS gas-flow measurement. For a given sensing structure, the heating power, gas velocity, and overheat temperature difference between the heater and ambient can be described by a King-type semi-empirical relationship [13],

(1) $$P=\left(A+B\cdot U^{n}\right)\cdot \Delta T$$

where A and B are empirical coefficients related to the sensing structure, gas properties, and heat-transfer state; U is the gas velocity; n is an empirical exponent; and ΔT is the heater-to-ambient overheat temperature difference. Heat-loss sensors can operate in constant-power (CP) or CTD modes, as illustrated in Figure 1a. In CP mode, P is held constant and the variation in ΔT is used to represent the flow. In CTD mode, ΔT is maintained at a fixed value and the heating power is adjusted through closed-loop control, resulting in a monotonic variation in the heater voltage or power with flow speed. Considering that the contamination experiments required repeated chip replacement and recalibration under different operating conditions, CTD operation was selected to reduce the influence of ambient-temperature variations on the reference temperature difference.

The chip layout is shown in Figure 1b. Wider metal segments were used in the interconnect regions to reduce electrical resistance and Joule heating outside the active sensing area, concentrating heat generation near the central heater and minimizing thermal coupling to the ambient-temperature resistor. The operating state of the chip was analyzed in COMSOL Multiphysics 6.2. The electrically active region was first simplified according to the actual package geometry, and the Electric Currents module was used to identify the locations of the main voltage drop and Joule heating. Laminar Flow and Heat Transfer in Solids and Fluids were then coupled through a non-isothermal-flow interface to solve the flow and temperature fields. Under CTD operation in Figure 1c, the overheat temperature difference was set to 20 °C; the thermal field generated by the heating resistor has a negligible effect on the ambient temperature sensing resistor.

In fabrication as shown in Figure 1d, a double-side-polished ceramic wafer was chosen as the chip substrate. First, a central Pt resistor served as the heater, while peripheral Pt resistors measured the ambient temperature. Integrating in a chip can reduce temperature-coefficient mismatch caused by material and process variations. Then, Au was utilized for pads to realize low-resistance interconnects. Finally, the device surface was protected using SiO_2_ passivation process with openings at the bonding pads. The designed resistances of the heater and ambient-temperature resistor were approximately 50 Ω and 1.5 kΩ, respectively. Figure 1e gives the photo of the fabricated flow sensor. After fabrication, the chip was wire-bonded to the external PCB circuit. The pads and wire-bonding region were sealed with silicone so that the active sensing area remained exposed while the electrical connections were protected, as shown in Figure 1f.

### 2.2. Curved Bypass Channel Method

Before reaching the sensing region, particles are transported through the main channel and the bypass channel. The bypass channel is arranged beside the main channel to reduce the local velocity over the chip and extend the measurement range. The flow-split ratio was defined as the ratio between the bypass flow rate and the main-channel flow rate,

(2) $$\beta =\frac{Q_{b}}{Q_{m}}\times 100\%\;$$

where *Q_m_* and *Q_b_* are the flow rate in main channel and bypass channel, respectively.

**Figure 2:**
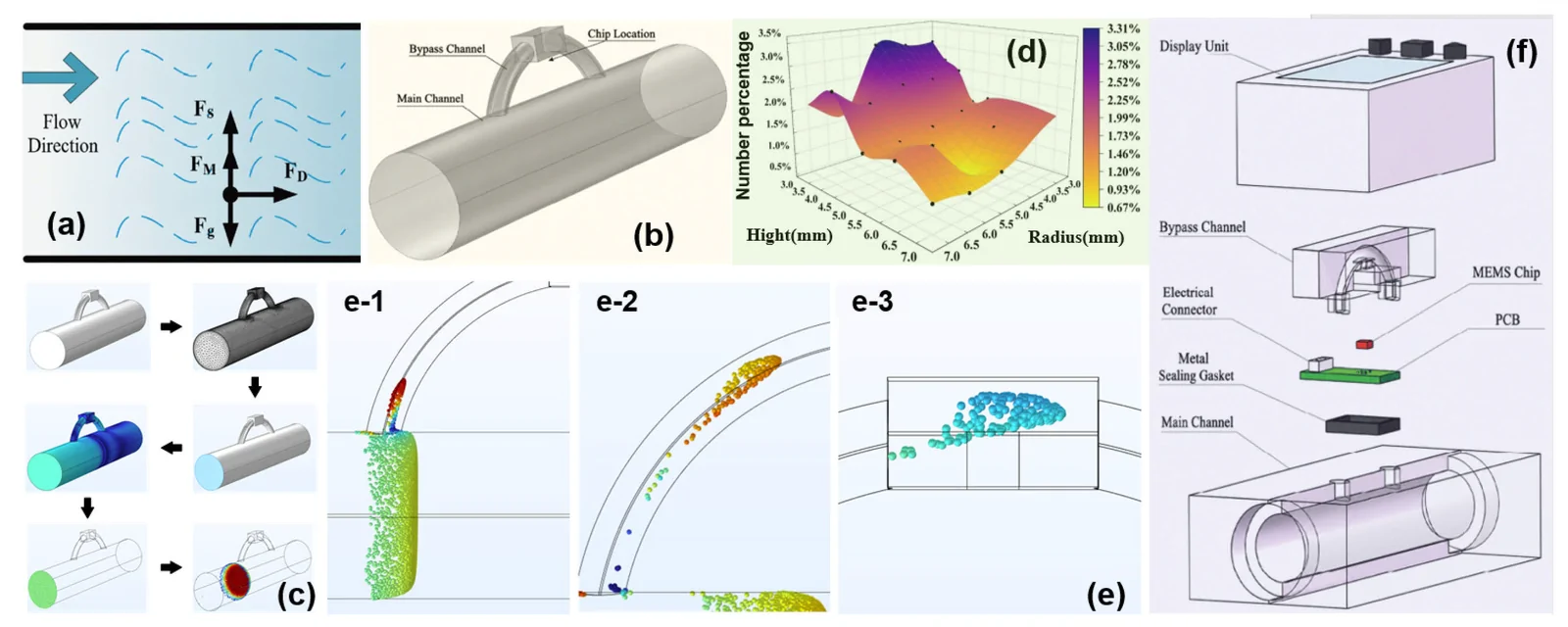
Curved bypass channel design, numerical model, and experimental setup. (**a**) Particles subject to force, (**b**) overall sensor/bypass structure, (**c**) air-domain numerical model, (**d**) Percentage of particles to the bypass channel, (**e1**–**e3**) particles number to the bypass channel, and (**f**) curved channel assembly to main channel.

A conventional straight bypass channel provides flow division but does not introduce a geometric mechanism that continuously drives particles toward one side of the cross-section. Particles in the channel are primarily subjected to aerodynamic drag, Saffman shear lift, gravity, buoyancy, and rotation-related forces. The principal force directions are illustrated in Figure 2a, and the motion of an individual particle can be written as

(3) $$m_{p}\cdot d\frac{v_{p}}{dt}=FD+FS+Fg+FM+Fother$$

where *m_p_* and *v_p_* denote the particle mass and velocity, respectively. FD, FS, Fg, FM and Fother are the fluid-induced drag force, Saffman lift force, gravity force, Magnus force and other force, respectively. For micro-scale spherical particles at low particle Reynolds number, the aerodynamic drag can be approximated by the Stokes expression,

(4) $$F_{D}=3\pi \;\mu \;d_{p}\left|u-v_{p}\right|$$

where μ is the dynamic viscosity of air, d_p_ is the particle diameter, and *u* is the local gas velocity. In a straight channel, particles predominantly follow the axial flow. When the bypass channel is curved, inertia prevents particles from exact tracking of the bending streamlines, causing gradual migration toward the outer side. Curved channels also induce Dean secondary flow, whose characteristic cross-sectional vortices are illustrated in Figure 2b and whose strength can be represented by the Dean number [14,15],

(5) $$De=Re\cdot \sqrt{\frac{D_{h}}{R_{c}}}$$

where *Re* is the Reynolds number, *D_h_* is the hydraulic diameter, and *Rc* is the channel curvature radius. Therefore, the curved bypass channel must balance lateral particle migration, secondary flow, inlet geometry, and flow-split stability. An Euler–Lagrange approach was used, in which the air flow was solved first as the continuous phase and discrete particle trajectories were then tracked. The air-domain model is shown in Figure 2c, with relatively refined meshes at the bypass inlet, curved section, and chip-mounting region. The simulated particles matched the silica fume used experimentally, with a diameter of 1 μm and density of 2200 kg/m^3^. A representative volumetric flow rate of 20 SLPM was used. Parametric sweeps covered r = 3–7 mm and the geometrically allowable range of h. Figure 2d gives the percentage of particles to the curved channel with different size. As shown in Figure 2e, a velocity gradient field exists in the main channel with a circular cross-section and the fluid velocity at the channel center is indeed higher than that near the boundary. Contaminant particles keep moving inside the bypass channel and gradually shift outward under the effect of particle inertia and secondary flow. An initial edge-concentrating effect can be observed clear in Figure 2e. The results demonstrate that modifying the structural design of the arcuate bypass channel alters particle trajectories, thereby reducing the distribution of contaminant particles on the surface of the sensor chip. It can effectively mitigate the impact of contaminant particles on the flow sensor. Figure 2f gives the bypass channel attached to the main channel. Curved and straight control housings were fabricated as integrated main channel/bypass structures by 3D printing to minimize leakage from assembly gaps.

### 2.3. Surface Electric-Field Regulation Method

**Figure 3:**
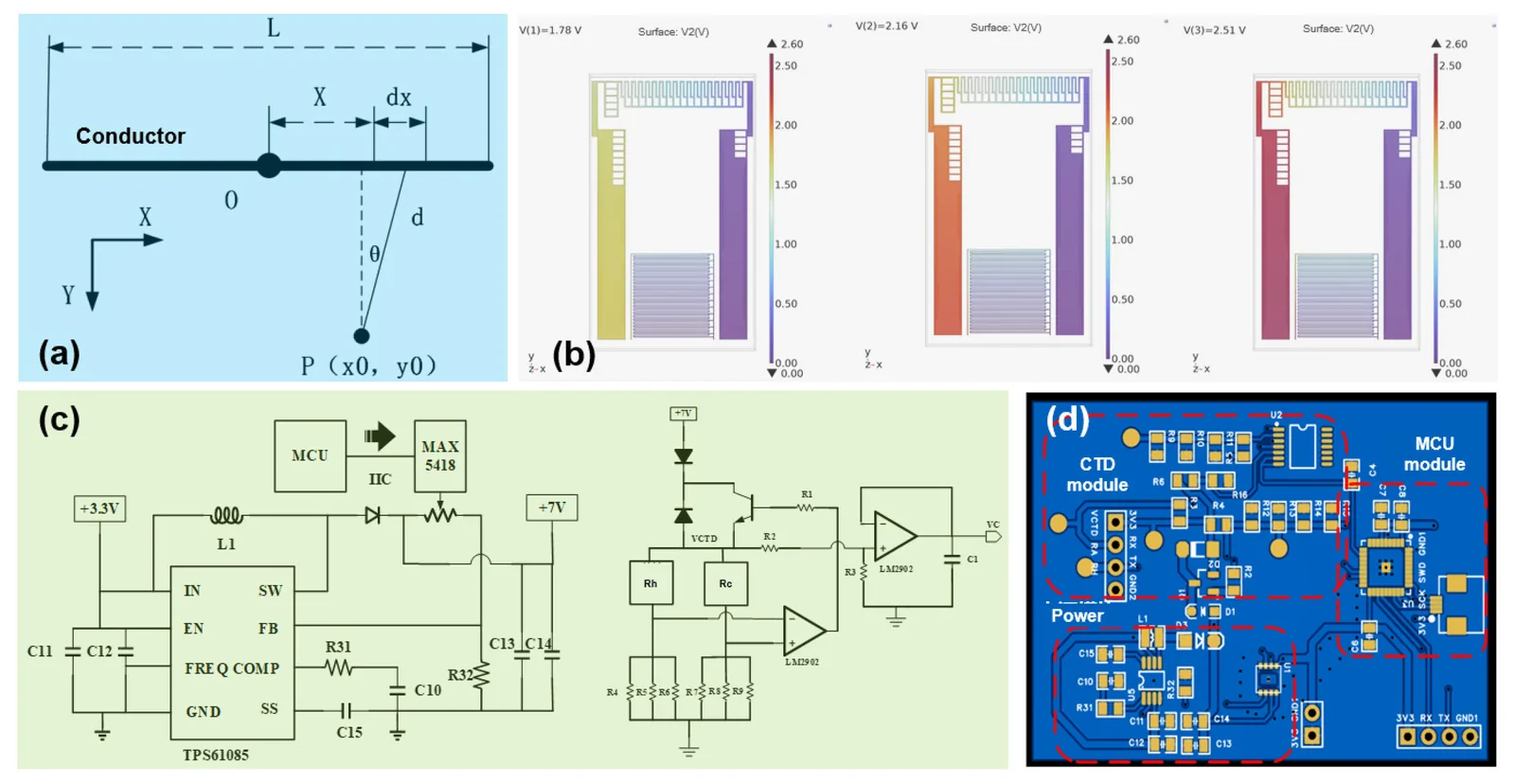
Surface electric-field regulation. (**a**) Electric-field force on the particle, (**b**) electric-field simulation by FEM, (**c**) adjustable power-supply module and (**d**) PCB prototype.

The curved bypass channel can reduce the particle load reaching the chip region, but a fraction of the particles can still enter the vicinity of the energized resistors. In Figure 3a, particles may acquire net charge through collision and friction, and once they enter the near-surface electric field, they experience an electrostatic force,

(6) $$F_{E}=q_{p}E$$

where q_p_ is the electric charge of particle and the E is the electric field. The heater and temperature-sensing resistors on the chip were approximated as an array of finite-length line electrodes, as illustrated in Figure 3b. For fixed geometry, increasing the conductor potential strengthens the electric field at a given particle location. Therefore, operating voltage can be used as a near-field control variable without changing the chip structure. The wider metal interconnects exhibit only small potential variations, while the major voltage drop is concentrated in the active Pt resistor region, indicating that the wide segments primarily provide electrical connection rather than forming Joule-heating sources comparable to the sensing region. As shown in Figure 3b, when the external supply voltages were 7, 8, and 9 V, the actual voltages across the chip resistors is approximately 1.78, 2.16, and 2.51 V, respectively. These values were used as the finite-element boundary conditions to compare field distributions and field intensity at identical reference locations. Reducing the operating voltage weakens the near-surface field, but it also compresses the output-voltage difference between adjacent flow-calibration points. To integrate field regulation into the original measurement electronics, in this paper, an adjustable power-supply module and MCU control were added to the CTD circuit, as shown in Figure 3c. A digital potentiometer at the boost-converter feedback node was controlled by the MCU through I^2^C interface, while serial communication was utilized for calibration, data transmission, and operating-mode switching. The voltage-control module in the PCB is shown in Figure 3d, enabling switching between normal measurement and the low-voltage anti-contamination operation, with a switching time of several milliseconds.

## 3. Results

### 3.1. Experimental Setup

**Figure 4:**
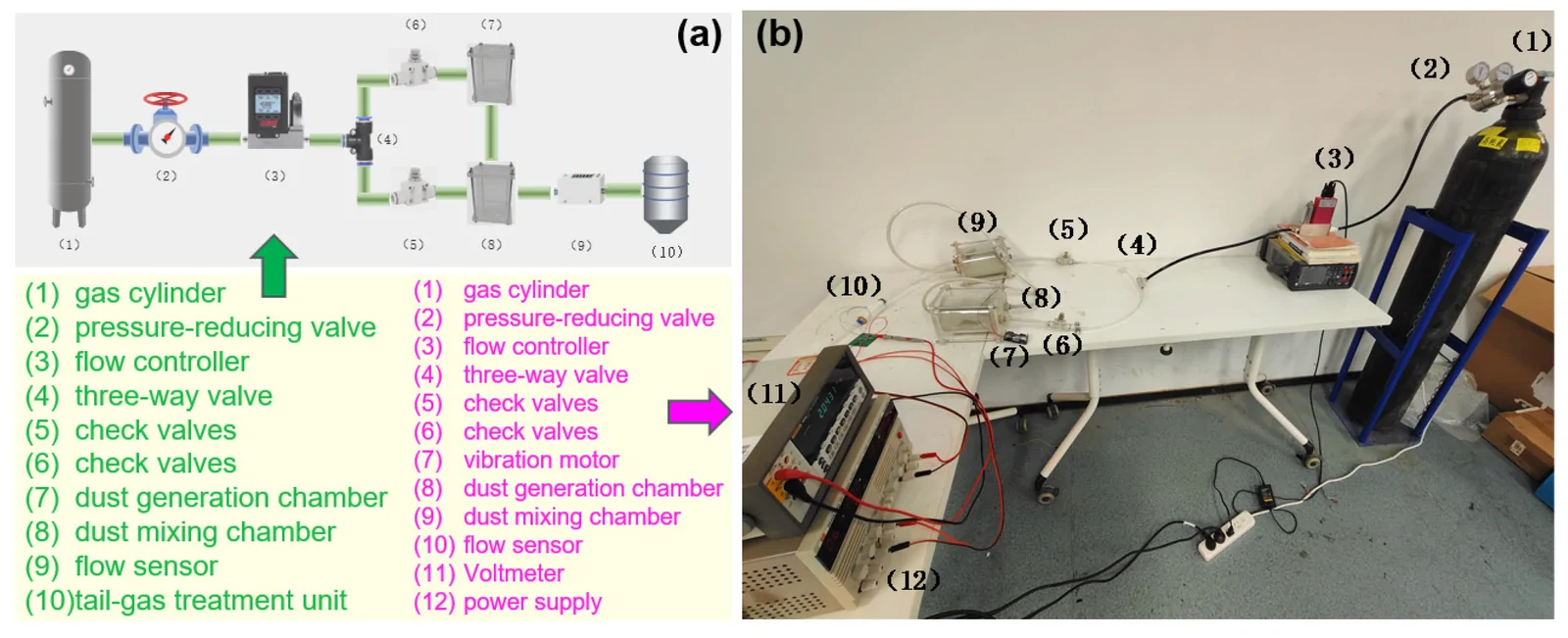
Flow experiments in particle environments. (**a**) Schematic of the setup and (**b**) photo of the test platform in the lab.

In this work, the silica fume was adopted to simulate the particles, and its diameter is about 1 μm, which is consistent with that in the simulation. Once aerosolized by a vibration motor, the silica fume can be distributed relatively uniformly within the gas flow. Figure 4a shows the experimental setup, including gas cylinder, pressure-reducing valve, flow controller, three-way valve, check valves, dust generation chamber, dust mixing chamber and tail-gas treatment unit, and Figure 4b gives the photograph of the test platform in the lab. A check valve is used to control the on/off of solid particulate contaminants and prevent backflow of gas flow from interfering during the experiment. The three-way valve and check valve divide the entire experimental gas flow path into two branches. The dust generator is employed to aerosolize particulate contaminants and fully mix them with the gas flow. In this experiment, a vibration motor drives the dust generator to sufficiently disperse the silica fume before mixing it with the gas flow, to achieve a stable supply of dust-laden gas flow with the particle concentration of 0.442 g/m^3^.

### 3.2. Curved-Bypass Particle Transport and Anti-Contamination Performance

**Figure 5:**
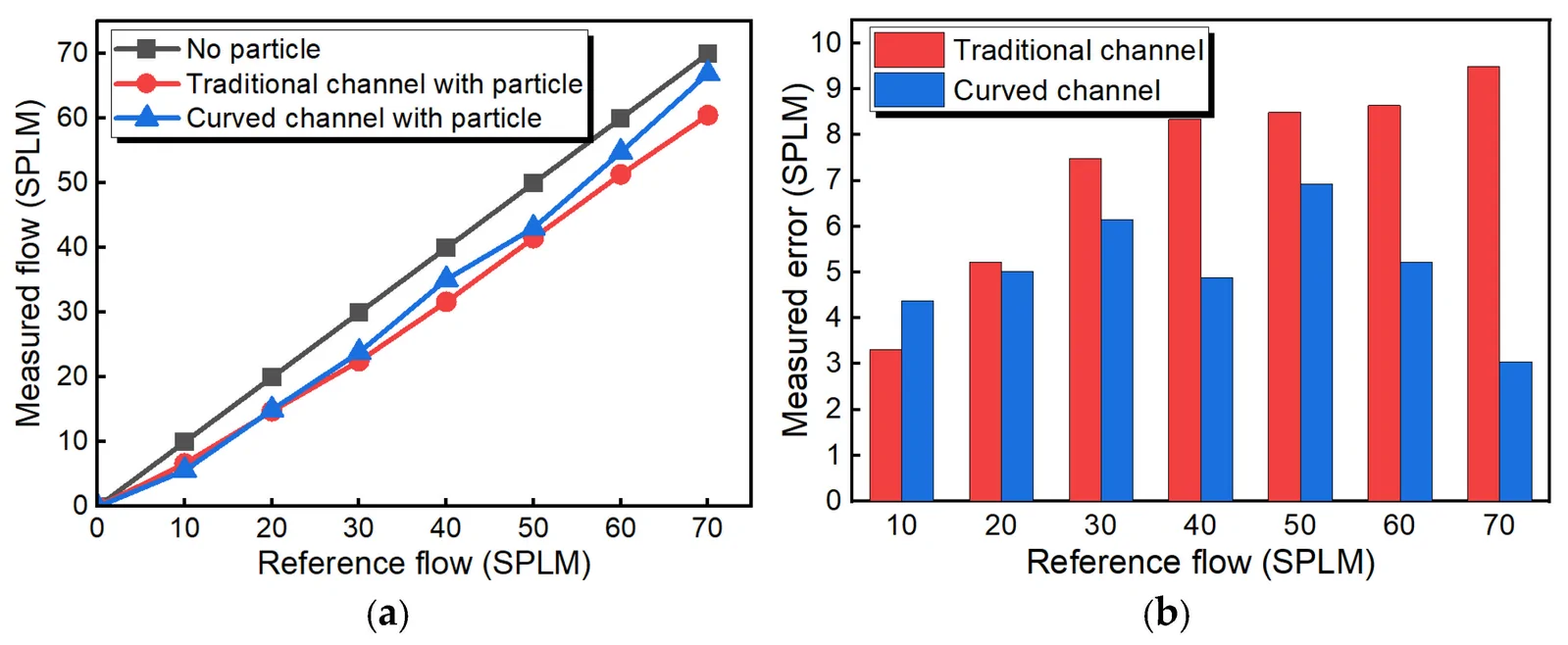
Experimental anti-contamination performance of the curved bypass channel. (**a**) Measurement results and (**b**) measurement errors.

The influence of the curved bypass channel was investigated under different flow speeds with particles. Because of the difficulty of flow-split ratio measurement, the length and the width of curved bypass channel are designed according to that of the traditional channel. Figure 5a indicates that in both the conventional straight channel and curved bypass channel, the sensor outputs are both degraded when the particle contamination exists in the air flow. Specially, at low flow rates (10–20 SLPM), the output difference between the two structures is small. This is mainly because the gas velocity is low, and particles are subjected to limited centrifugal force under such conditions. Hence, the curved channel cannot effectively change the particle distribution. As the flow rate increases to above 30 SLPM, sufficiently high gas velocity gradually intensifies the outward migration of particles inside the curved channel. This reduces the particle concentration in the sensing region and endows the sensor with superior anti-contamination performance. At high flow rates (70 SLPM), this difference is further enlarged. The error is defined as the difference of measured flow speed between the situation of no particle and that with the curved channel (or the traditional channel). Hence, compared with the conventional straight-channel structure, the curved bypass channel reduces the measurement error induced by particle contamination by approximately 22.5% on average for the flow speed up to 70 SPLM in Figure 5b, which verifies its anti-contamination advantage under medium-to-high flow-rate conditions. Consequently, the curved bypass channel design possesses favorable anti-particle-contamination capability, which becomes more prominent at high gas flow velocities.

**Figure 6:**
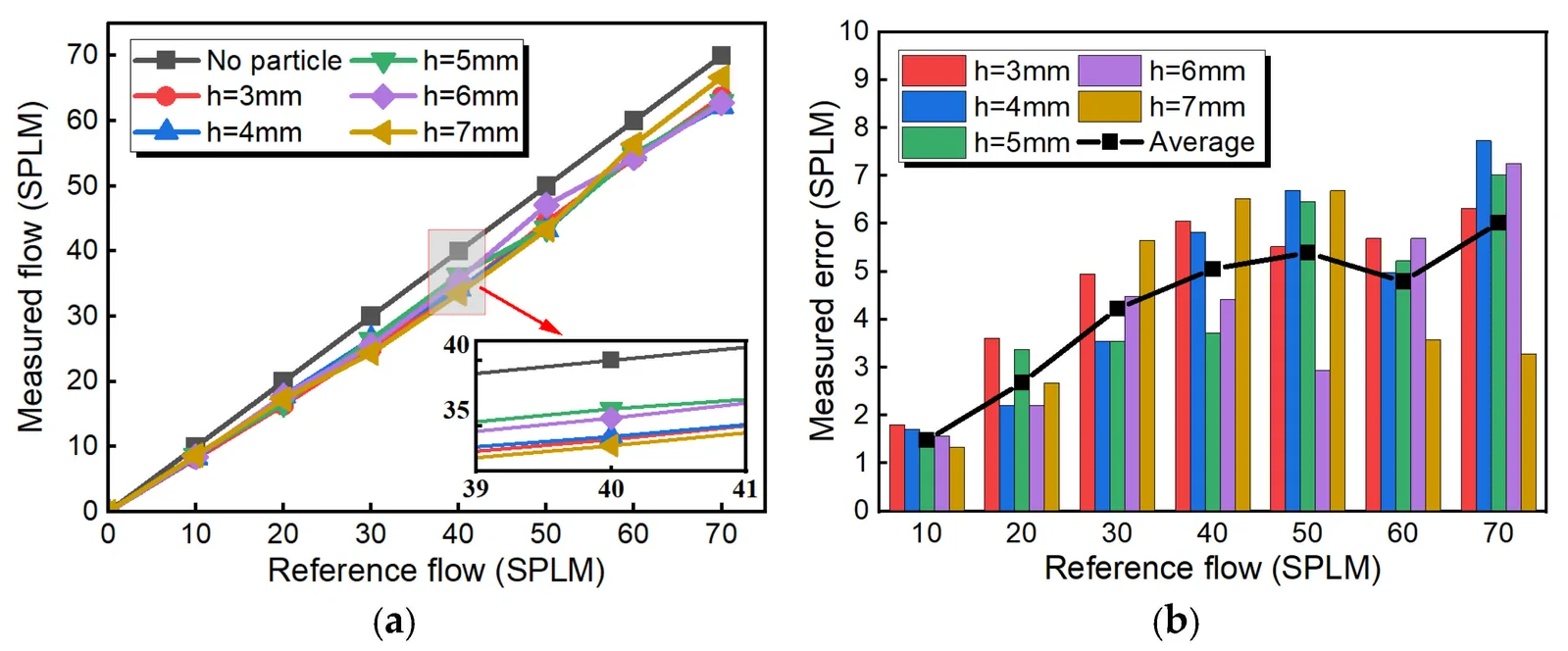
Channel height influence on the anti-contamination of the flow sensor. (**a**) Measured flow speed up to 70 SPLM and (**b**) tested errors.

The height dependence was further assessed by fabricating curved bypasses with r = 5 mm and h = 3–7 mm and subjecting them to the same contamination protocol. As shown in Figure 6a, the tested results show that the thermal flow sensor with an arcuate bypass channel exhibits an approximately linear increase in output flow against the reference flow, with good linearity, and all errors remain within a small range. As the height of the arcuate bypass channel increases from 3 mm to 7 mm, the error between the output flow and reference flow decreases in Figure 6b. However, for the sensor with a channel height of 7 mm, the output flow error rises abnormally to some extent, which is consistent with the simulation results presented earlier. The error values for arcuate bypass channels of different heights show obvious discrepancies at high flow rates. Meanwhile, the abnormal rise in output error of the 7 mm-height sensor is prominent in the range of 30–50 SPLM.

**Figure 7:**
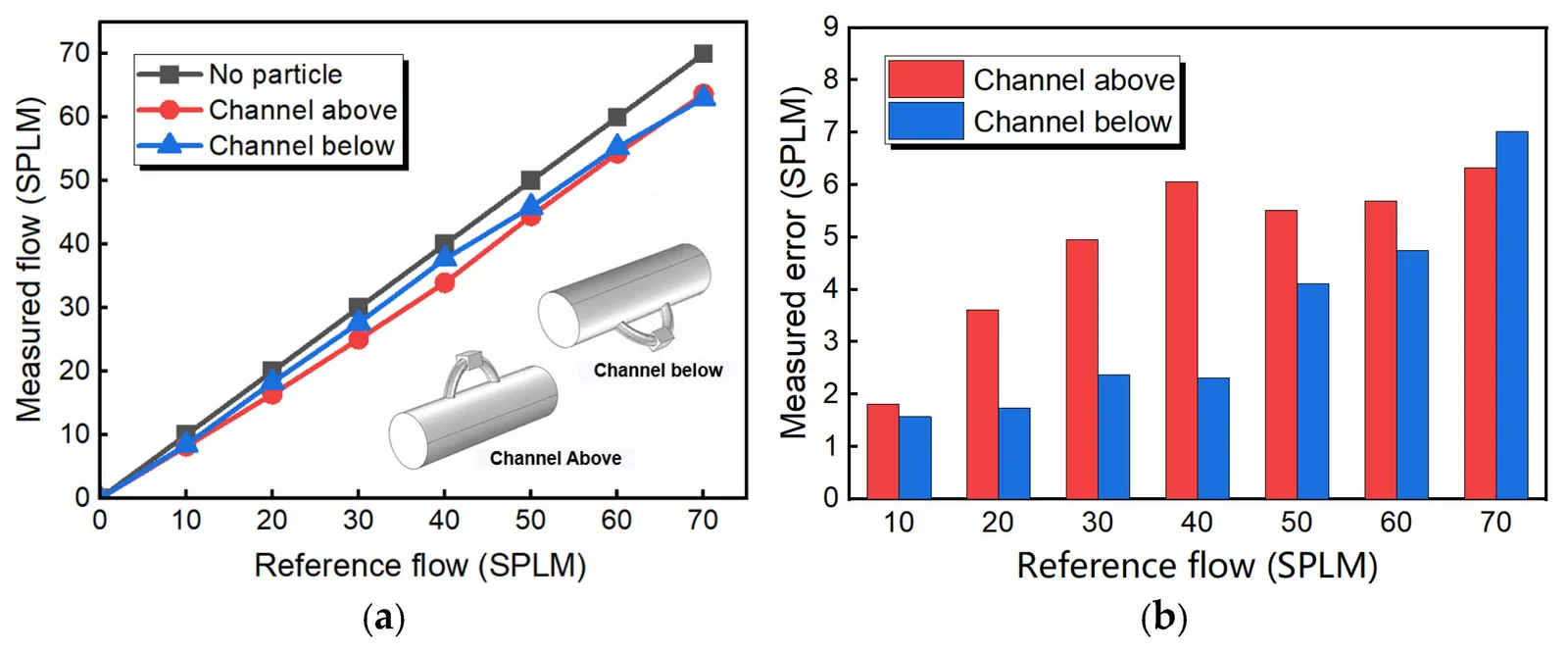
Gravity influence for the curved bypass channel. (**a**) Measured results and (**b**) measured errors.

Moreover, the effect of sensor orientation was evaluated by inverting the housing so that gravity acted in the opposite direction relative to the curved bypass, as illustrated in Figure 7. The experimental results demonstrate that at low flow velocities, the gravity effect forced the solid contaminant particles to migrate further toward the outer side of the arcuate bypass channel, yielding improved resistance to solid particle contamination. However, as the gas flow velocity increases, the particle inertia and secondary flow gradually dominated. Even without flipping the sensor housing, solid contaminant particles move closer to the outer wall of the arcuate bypass channel. Under such conditions, gravity exerts a relatively limited effect on the solid-particle contamination resistance of the sensor system.

### 3.3. Electric-Field Regulation and Surface Particle Deposition

**Figure 8:**
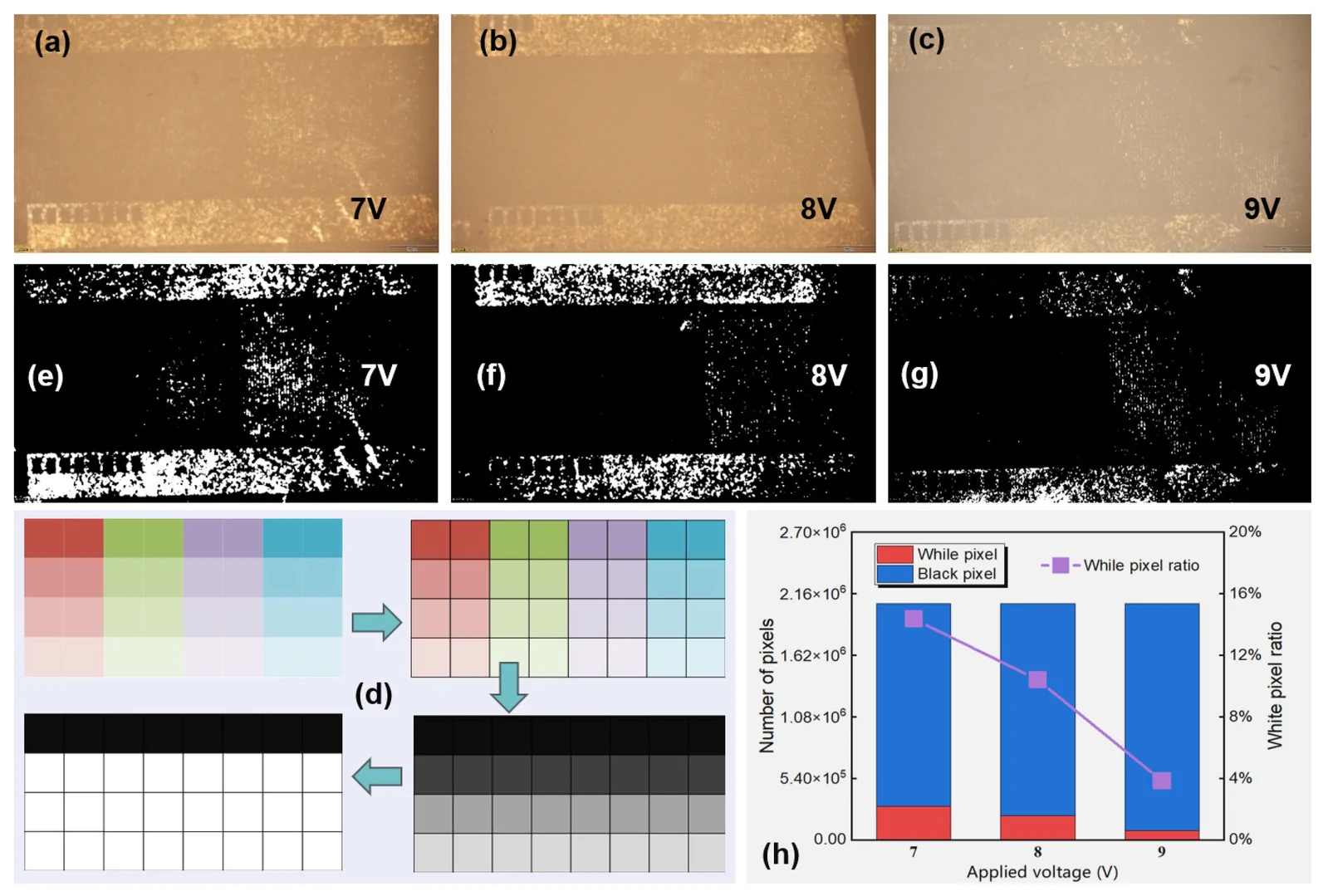
(**a**–**c**) Particle deposition at different operating voltages, (**d**) schematic diagram of the quantification process, (**e**–**g**) schematic diagram after processing at different voltage, and (**h**) schematic diagram of pixel fraction.

To verify the effectiveness of the electric-field regulation method, long-duration particle-contamination tests were conducted using the setup in the lab. The sensor was tested with the flow rate of 10 SLPM for 2 h under different supply voltages. Figure 8a–c shows the micro photo of the chip surface with applied voltages of 7 V, 8 V and 9 V, respectively. Although the contaminant particles and the ceramic substrate of the chip can be effectively distinguished, and the gradual increase in particle deposition from top to bottom can be visually observed, quantitative analysis methods are still lacking. To address this issue, this work proposes a threshold segmentation method using OpenCV (Open Source Computer Vision Library) for quantification. To avoid relying solely on qualitative visual assessment, all images were processed using a common segmentation procedure as shown in Figure 8d. Each image had a resolution of 1920 × 1080 pixels, corresponding to 2,073,600 pixels in total. Figure 8e–g shows the image obtained by converting Figure 8a,b to grayscale and performing threshold segmentation. The calculation results verify that the white pixel fractions in the three images are about 14.34%, 10.40%, and 3.84%, as presented in Figure 8e. A smaller white pixel fraction corresponds to a larger area of the chip surface covered by the contaminant particles. Compared with the condition at the highest voltage of 9 V, the coverage ratio of contaminant particle deposition on the sensor chip surface decreases by 10.5% in absolute terms at the lowest voltage of 7 V among the three tested voltages. Therefore, reducing the actual supply voltage of the chip contributes significantly to mitigating the contaminant particle deposition on the chip surface.

## 4. Discussion

From the perspective of contamination development, output drift in a thermal flow sensor arises from progressive changes in local flow and heat-transfer boundaries caused by particles. A sensor operated in CTD mode first provides a reference in clean gas. Once particles enter the bypass, whether they reach the active area depends on their transport trajectory through the branch. Even if a fraction of particles continues into the chip vicinity, the final deposition state can still be influenced by the electric field generated by the energized resistors. Consequently, channel geometry and operating voltage affect two successive stages of the same contamination process, and both ultimately manifest through changes in the flow–output relationship.

The effectiveness of the curved bypass depends on the combined action of particle inertia, Dean secondary flow, inlet geometry, and flow-split characteristics rather than on curvature alone. Pronounced outward migration was observed for relatively small curvature radii and moderate heights, whereas excessive inlet angles shifted the initial particle-entry position and weakened the benefit. In addition, some geometries with low particle fractions did not maintain stable flow-split ratios. This behavior is consistent with recent studies of coupled migration in curved and hybrid channels [16,17]. For a flow sensor, structural optimization should account simultaneously for particle avoidance and stable flow division. Otherwise, apparent contamination improvements can be confounded with changes in the underlying calibration relationship.

Admittedly, lowering the voltage may reduce the measurement sensitivity for the flow sensor. Consequently, a trade-off should be taken into consideration for different applications. Lower voltage reduces the chip near-surface electric field and, in the long-duration tests, is associated with a larger uncovered or lightly contaminated area. At the same time, reducing the supply voltage compresses the output-voltage spacing between adjacent calibration points. Electric-field regulation is therefore more suitable as an operating-state variable than as a single fixed low-voltage setting. Recent MEMS thermal flow sensors have used multimode readout, intelligent estimation, and structure-algorithm co-design to extend their effective operating range [3,6]. The present system similarly allows the control electronics to participate in adaptation to particle-laden conditions. The near-surface field can be reduced when contamination risk is high, while normal operation can restore greater output separation for routine measurement.

It should also be recognized that contamination-induced CTD output drift is not equivalent to a change in a single surface-coverage metric. Deposited particles can modify the near-wall velocity distribution and surface roughness and can alter the effective heat-transfer coefficient between the heater and gas through their own thermal properties. Because the CTD loop actively maintains a fixed overheat temperature, these boundary-condition changes are translated into variations in heating power and output voltage. Accordingly, the curved-bypass experiments use flow-measurement error to evaluate transport-stage control, whereas the electric-field experiments use optical surface coverage to evaluate deposition-stage changes. These metrics characterize different physical stages of contamination, while both relate to long-term sensor-output stability.

Table 1 summarizes the comparison between the proposed method and state-of-the-art technologies. From an engineering perspective, both control strategies are embedded directly in the existing sensor architecture. The curved bypass channel is obtained by reshaping the original flow-dividing branch and does not require a separate filtration unit, while electric-field regulation is implemented through the existing power and control chain without modifying the active sensing structure. The experiments further demonstrate that the two mechanisms are most influential under different operating conditions. The improvement from the curved bypass is more pronounced at medium and high flow rates, whereas the operating voltage directly changes the near-surface field and the deposition state after 2 h of exposure. Future studies that consider particle-size distributions, particle charge, humidity, and longer accumulation periods may clarify the optimum matching between channel geometry and operating state for different contamination environments.

## 5. Conclusions

A ceramic-substrate Pt MEMS thermal flow sensor operating in CTD mode was designed and implemented for particle-laden gas environments. At the overheat temperature difference of 20 °C, the heating and ambient-temperature regions were effectively thermally isolated, and the required heating power increased monotonically with flow velocity in agreement with heat-loss theory. Replacing the conventional straight bypass with a curved branch caused particles to migrate progressively toward upper half of the channel. In particle-laden tests, the curved bypass reduced the average contamination-induced measurement error by approximately 22.5% relative to the straight configuration, with stronger improvements at medium and high flow rates. Operating-voltage regulation was subsequently adopted to adjust the chip surface electric field, indicating that lower operating voltage mitigated surface deposition. By integrating particle-transport control before arrival at the chip with near-surface deposition control after arrival, the proposed design provides an integrated route for improving the long-term stability of MEMS thermal flow sensors in particle-laden environments.

## Figures and Tables

**Table 1:** Comparison of anti-particle contamination for flow sensors.

Reference	Method	Size	Disadvantage
[18]	Burn out	Small	High temperature
[19,20]	Thermophoretic force	Small	High temperature
[21]	Cyclone structure	Large	Large in volume
This work	Bended bypass and adjustable voltage	Small	-

## Data Availability

The data presented in this study are available on request from the corresponding author.
